# Tectonics of earthquake swarms in the Tokara Gap of northern Ryukyu Arc based on marine geological and geophysical surveys

**DOI:** 10.1038/s41598-026-41371-z

**Published:** 2026-04-18

**Authors:** Hiroaki Koge, Saki Ishino, Yumiko Harigane, Ayanori Misawa, Taichi Sato, Osamu Ishizuka, Gen Shimoda, Jun Arimoto, Seishiro Furuyama, Chiori Tamura, Hiroki Minami, Takaaki Tamai, Suguru Yabe, Yuta Amezawa, Hajime Katayama, Yoshiaki Suzuki, Mikiya Yamashita, Seitaro Ono, Yuka Yokoyama, Takami Mori, Yusuke Sato, Katsura Kameo, Ryosuke Komatsu, Mako Nakao, Takahiko inoue

**Affiliations:** 1 Geological Survey of Japan, National Institute of Advanced Industrial Science and Technology (AIST), 1-1-1 Higashi, Tsukuba, 305-8567 Ibaraki Japan; 2 Marine Resources and Energy, Tokyo University of Marine Science and Technology, 4-5-7 Konan, Minato-ku, Tokyo, 108-8477 Japan; 3 Atmospheric and Ocean Research Institute, University of Tokyo, 5-1-5, Kashiwanoha, Kashiwa, 277-8581 Chiba Japan; 4 Japan Coast Guard, 3-1-1 Kasumigaseki, Chiyoda-ku, Tokyo, 100-8932 Japan; 5 School of Marine Science and Technology, Tokai University, 3-20-1 Orido, Shimizu Ward, Shizuoka, 424-8610 Japan; 6 Marine Works Japan Ltd, 54-1 Oppamahigashicho, Yokosuka, 237-0063 Kanagawa Japan; 7 MOL Marine & Engineering Co. Ltd. Ltd, Shosen-Mitsui Bldg., 2-1-1, Toranomon, Minato-ku, Tokyo, 105-0001 Japan; 8 Present Address: MOL Maritex Co. Ltd, Shosen-Mitsui Bldg., 2-1-1, Toranomon, Minato-ku, Tokyo, 105-0001 Japan

**Keywords:** Ryukyu Arc, Tokara Gap, Seismic swarm, RRR triple junction, Geophysical observations, Natural hazards, Solid Earth sciences

## Abstract

The 2025 Tokara seismic swarm, which began in late June, has been the largest documented sequence of earthquakes in the northern Ryukyu Arc. It has generated more than 2200 earthquakes, including episodes of strong ground-shaking at Akusekijima Island. The mechanisms responsible for generating repeated swarm activity in the Tokara Gap remain poorly understood. The most recent event, characterized by very frequent and intense shaking, rapidly amplified societal impacts. To investigate the tectonic framework of this area, we analyzed marine geological and geophysical datasets, including multibeam bathymetry, multichannel seismic profiles, marine magnetic anomalies, and Bouguer gravity anomalies. Seafloor morphology and seismic profiles revealed two intersecting extensional fault systems: a WNW–ESE set and an ENE–WSW set. Localized magnetic and topographic highs suggested shallow magmatism. Previous geochemical studies have indicated that hydrothermal plumes along the volcanic front, near the western swarm area, comprise a mixture of deep-sourced fluids and volcanic-front components. However, water-column surveys after the December 2021 swarm detected no persistent hydrothermal plumes. While the relative roles of magma and fluids remain uncertain, the evidence suggests that the 2025 swarm reflects processes typical of incipient back-arc rifts, where episodic magmatic intrusion and fluid overpressure interact.

**Supplementary Information:**

The online version contains supplementary material available at 10.1038/s41598-026-41371-z.

## Introduction

The Tokara Islands in southwestern Japan experienced recurrent seismic swarm activities in 1995, 2000, and 2021^1,2^. Each swarm typically lasted several weeks and involved several hundred to a few thousand earthquakes. The 2025 Tokara Gap (TG) swarm was exceptional in terms of both duration and seismic productivity. The more than 2200 earthquakes recorded between 21 June and 4 August exceeded the number recorded in any documented sequence. The activity intensified in late July and culminated in an intensity of 6 Lower (Japan Meteorological Agency [JMA] intensity scale) on Akusekijima Island on 30 July. This intensity corresponds to a magnitude of ~ 5.2 on the Moment Magnitude Scale based on the F-net centroid moment tensor solution^3^. Public reports from the JMA and ERC indicated that the temporal evolution of the sequence, which was marked by sustained, high activity that escalated in late-July, differed markedly from that of earlier swarms. Analysis of the 2025 series of swarms therefore provided an opportunity to better understand swarm-generating mechanisms in the northern Ryukyu Arc.

At convergent plate boundaries, seismicity includes not only megathrust earthquakes along the plate interface but also inland, outer-rise, and intraslab earthquakes associated with deformation of the overriding and subducting plates. The Nankai Trough and Japan Trench represent well-known settings where these diverse earthquake types coexist. Back-arc rifting regions such as the Japan Sea, Okinawa Trough, and the Izu–Bonin arc exhibit deformation patterns different from those typical of convergent-margin tectonics.

In Japan, long-lasting, high-frequency earthquake swarms confined to a narrow area are relatively rare in both typical convergent-margin tectonic settings and back-arc rifting systems. One of the few recognized analogues in Japan is the repeated swarm activity beneath the Izu Peninsula (e.g.,^4^. The ongoing swarm in the Tokara Gap therefore represents an exceptionally unusual style of seismicity.

**Fig. 1:**
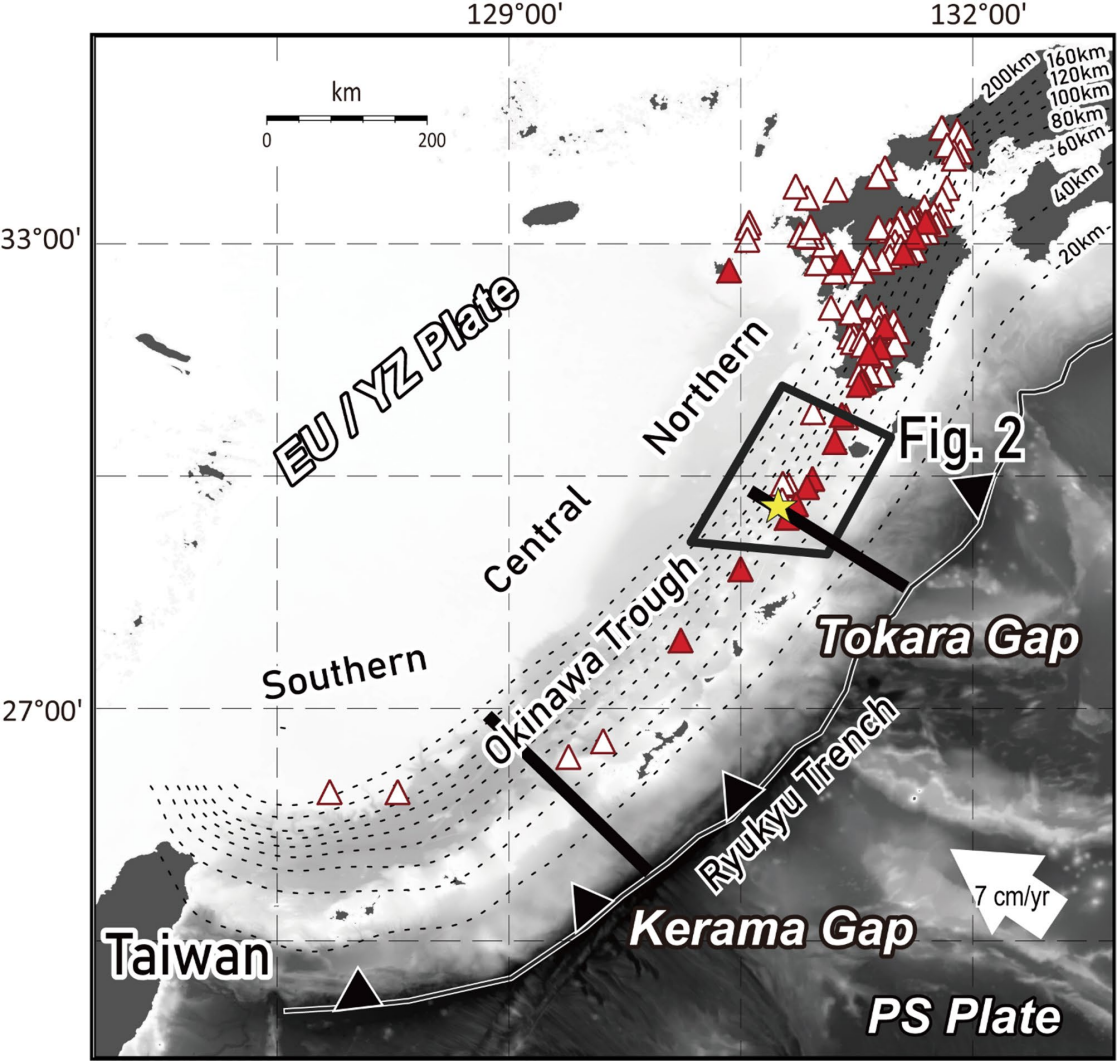
Regional tectonic setting, bathymetry, and fault system classification of the Tokara region. Tectonic map of Okinawa Trough and location of study site in the Tokara region (black quadrilateral). Red triangles indicate active volcanoes listed by the Japan Meteorological Agency, and white triangles denote submarine and terrestrial volcanoes compiled by the Geological Survey of Japan National Institute of Advanced Industrial Science and Technology. The yellow star marks the main position of the 2025 seismic swarm (e.g., the F-net seismograph network;^3^. The velocity of the Philippine Sea (PS) plate was calculated with the NNR-MORVEL56 model^5^ with the Yangtze (YZ) plate held fixed. A calculation with the Eurasian (EU) plate fixed gave nearly the same result. Slab depth contours are derived from the Slab2 model^6^. This figure was drawn using QGIS v3.40^7^.

This ongoing swarm has resulted in significant societal impacts, including the evacuation of residents of the Tokara Islands (Fig. 1). These unusual swarm events underscore the urgent need to better understand the tectonic mechanisms driving seismic swarm activity in this region.

Two main possibilities have been tentatively proposed: tectonic extension associated with back-arc rifting, and involvement of magma or deep-seated fluids along the volcanic front. The Tokara region overlies the subducting Philippine Sea Plate along the Ryukyu Trench, where arc and back-arc processes interact to generate extensional deformation and associated active volcanism on the overriding Eurasian Plate (or Yangtze Plate). The Tokara Islands are embedded within a tectonically complex region characterized by multiple directions of the extension system and volcanic activity^8–11^. That the Tokara Islands are part of the Quaternary volcanic front of the Ryukyu Arc implies not only elevated seismic hazards but also the potential for volcanic unrest.

**Fig. 2:**
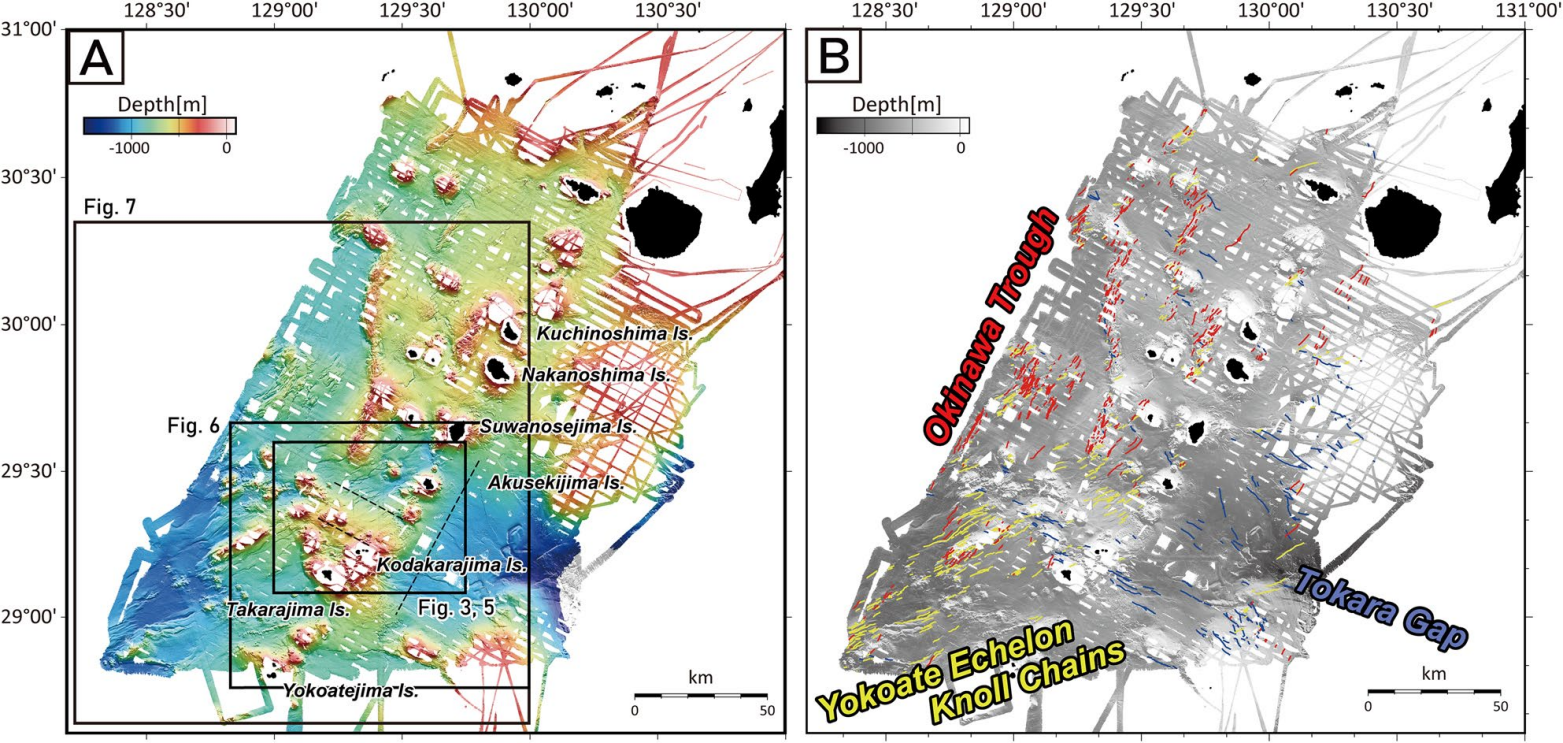
High-resolution bathymetry and fault system classification of the Tokara region. (**A**) Enlarged view of the Tokara region (black quadrilateral) showing a compilation of high-resolution bathymetric data gridded at 20-m intervals that were accumulated from previous research cruises^9^. Inner black rectangles correspond to detailed map areas referenced in subsequent figures. Dashed lines indicate multichannel seismic (MCS) profiles, with profile names labeled alongside each line. (**B**) Enlarged view of the Tokara region (black quadrilateral). Three groups of fault systems are shown: the Okinawa Trough (OT), the Tokara Gap (TG), and the Yokoate Echelon Knoll Chains (YE), with representative orientations of N52.97°E, N71.74°W, and N71.30°E, respectively^9^. (A) and (B) were drawn with GMT6^12^.

However, detailed and comprehensive marine geological and geophysical observations have remained limited. Between 2020 and 2023, the Tokara Islands were covered by marine geological and geophysical surveys conducted by the Geological Survey of Japan (GSJ), National Institute of Advanced Industrial Science and Technology (AIST), as part of compilation marine geological maps of the surrounding seas (Fig. 2). These surveys employed a high-density grid of survey lines. Lines across arcs (WNW–ESE) were spaced at ~ 3.7-km intervals, and lines parallel to arcs (NNE–SSW) were spaced at ~ 7.4-km intervals. Follow-up surveys were conducted in key areas that required further investigation. These surveys provided the first high-density datasets ever collected in this region. The surveys included bathymetry, seismic profiles, and magnetic data that enabled a more detailed understanding of the geological setting. We used these new datasets to elucidate the seafloor morphology and subsurface crustal architecture of the Tokara Gap and to explore the relationships between the ongoing seismic swarm and underlying tectonic and magmatic processes, including possible fluid involvement. Our findings provided insights into crustal deformation and seismicity within this tectonically transitional subduction region along a back-arc boundary.

### Geological background

The Ryukyu Arc, which extends ~ 1200 km between Kyushu and Taiwan (Fig. 1), formed because of subduction of the Philippine Sea Plate beneath the Eurasian Plate (or Yangtze Plate) along the Ryukyu Trench. This arc consists of the Ryukyu Trench, the Ryukyu Islands, a frontal arc, and the Okinawa Trough (OT) from east to west (Fig. 1^13^). The Ryukyu Arc is commonly divided into northern, central, and southern segments. The northern and central sections are separated by the Tokara Gap (~ 29.3ºN), and the central and southern sections are separated by the Kerama Gap (~ 26ºN) (Fig. 1; e.g.,^13–16^ have identified three phases of extension in the Okinawa Trough: the first phase during the Middle–Late Miocene, the second phase during the Early Pleistocene, and the third phase during the Late Pleistocene–Recent. Although these three phases constitute a simplified framework, other studies have emphasized more complex evolutionary scenarios involving shifts in motion of the Philippine Sea Plate and collision of the Ryukyu Arc and continent around Taiwan (e.g.,^17,18^ have given a more detailed review of Quaternary crustal deformation.

Along the Ryukyu Arc, there is a marked along-arc variation of Quaternary volcanic activity. In the northern segment, the Tokara Islands define a clear volcanic front; in the southern segment, the position of the front is less well defined and has been associated with submarine knolls such as Irabu, Tarama, and Kume (e.g.^19–21^). Geochemical data revealed an increase of sediment contributions toward the south^22^, which not only indicated a systematic along-arc gradient, but also suggested that subduction and back-arc opening processes had become established earlier in the southern segment than in the north.

The Tokara Gap (TG) represents a major tectonic and volcanic boundary within the northern Ryukyu Arc. A distinctive feature of this area is that Quaternary volcanic activity is expressed both along the frontal arc and along the parallel rear-arc chain (Fig. 1^11,23^). These volcanoes have formed from the early Pliocene to present^24–28^. This area has also been undergoing regional extensional deformation, particularly on its back-arc side.

Arai et al.^29^ have shown multichannel seismic (MCS) reflection data that identified a ~ 3-km-deep sedimentary basin beneath the TG. The indication is that there has been localized subsidence associated with rifting or arc-parallel extension. Their P-wave velocity section (see the RK02 profile in^29^) has also revealed significant thinning of the upper crust (5.0–6.0 km/s) and a continental Moho located at a depth of ~ 25 km beneath the TG. Arai et al.^29^ have also noted that the northern part of the subducting plate consists of large, buoyant oceanic plateaus, including the Amami Plateau, Daito Ridge, and Oki-Daito Ridge^30,31,21^. These plateaus may enhance the concentration of active volcanism on the overlying arc during subduction^32,33^.

Relevant to the structural complexity along the arc-facing slope has been the identification by Minami et al.^11^ of a series of cross-arc grabens around the Shirahama Bank with NW–SE to WNW–ESE orientations. This finding has been interpreted as evidence for an arc-parallel extension independent of the back-arc rifting observed in the OT. They have also revealed multiple volcanic edifices and active bubble plumes within these grabens based on high-resolution bathymetric and water-column data. Recent volcanism has therefore been structurally controlled by arc-parallel extensional processes.

Recent geomorphologic, magnetic, and seismic studies have confirmed that there are volcanic cross-arc chains in the northern OT that have been termed the “Yokoate Echelon Knoll Chains” (YE)^34,8^. Detailed bathymetric surveys in the Tokara region by Koge et al.^9^ have revealed numerous seafloor lineaments indicative of active faulting (Fig. 2). K-means clustering of their orientations has identified three principal fault systems corresponding to the OT, TG, and YE. Centroid Moment Tensor (CMT) analysis has indicated that normal faulting predominates in all three systems. Stress inversions have shown that these systems formed under a unified north–south extensional stress regime associated with rifting of the OT. The curvature of the Ryukyu Arc allows a single extensional stress field to generate extensions in two directions, which in turn results in three rifting axes that satisfy the conditions for formation of a ridge–ridge–ridge (RRR) type triple junction. These observations suggest that the present fault configuration may represent the inception of an apparent RRR triple junction. If the Okinawa Trough continues to expand, these fault systems will remain active and may lead to its full development.

## Results

### Morphological and structural features of the swarm earthquake region

#### Detailed bathymetric observations and lineament patterns

Previous studies have shown that the Tokara region is structurally complex, characterized by the intersection of three distinct extensional fault groups (Fig. 2B): the TG (blue, N71.74°W), YE (yellow, N71.30°E), and OT (red, N52.97°E)^9^.

**Fig. 3:**
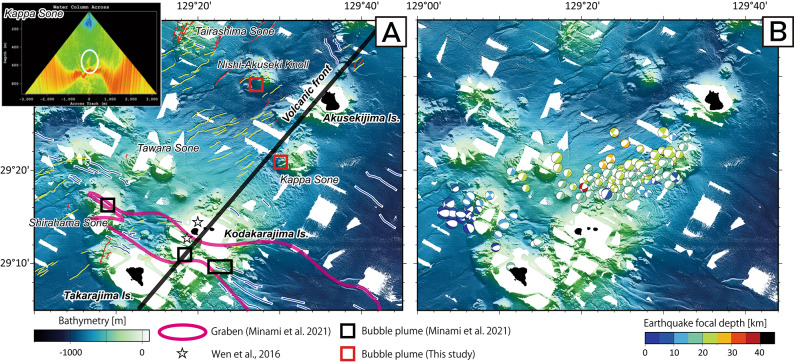
Spatial distribution of seafloor morphology, hydrothermal plumes, and seismicity in the Tokara region. (**A**) Bathymetric map. The pink-shaded area indicates the graben zone in the Tokara Gap described by Minami et al.^11^. Black square boxes show the locations of bubble plumes identified by Minami et al.^11^; white star symbols denote plume sites reported by Wen et al.^35^. Newly identified bubble plumes from the KH-22-2 cruise are shown as red square boxes, with representative water column images displayed in the top-left. The approximate location of the volcanic front is marked by a gray line. Colored lineaments correspond to the structural trends classified by Koge et al.^9^: red for the Okinawa Trough (OT), yellow for the Yokoate Echelon (YE), and blue for the Tokara Gap (TG). (**B**) Bathymetric map with superimposed centroid moment tensor (CMT) solutions for the 2025 swarm from the F-net catalog. (**A**) and (**B**) were drawn with GMT6^12^.

Building on these classifications, we conducted a detailed morphological analysis of the seismic swarm area. The TG lineament group corresponds to a well-developed graben structure^11^ along the TG (Fig. 3) and comprises a series of normal faults that extend from the volcanic front toward the back-arc region. In contrast, the YE lineament group is predominantly distributed on the western side of the volcanic front and is rarely observed to the east of it. The OT group is also present but is less dominant than the other two groups (Fig. 2B).

Several knolls and banks are scattered throughout the region. Features such as the Tairashima Sone (“sone” means bank in Japanese), Nishi-Akuseki Knoll, Tawara Sone, Kappa Sone, and Shirahama Sone are officially recognized names adopted by the Japanese Committee on Undersea Feature Names (JCUFN) (Fig. 3). The use of high-resolution bathymetric data with a 20-m grid has enabled the identification of much finer-scale seafloor features. A particularly noteworthy feature are the small knolls along the southern margin of the seismic swarm area, just north of Kodakarajima Island (around 29°17′N, 129°22′E). These knolls have not been recognized in previous surveys and may reflect localized volcanic or tectonic processes.

The OT, TG, and YE lineaments intersect near the seismic swarm area (Fig. 3). The highest concentration of swarm activity between 21 June and 21 July 2025 was located near this central intersection zone. The distribution of earthquake hypocenters, illustrated in Fig. 3, exhibited a distinct bimodal pattern. Given the sparse station coverage in the Tokara region, depth estimates based on CMTs are associated with large model-dependent uncertainties, and differences of depths should be interpreted with caution. The western cluster, located around Shirahama Sone, corresponded to the graben zone and were characterized by predominantly normal faulting consistent with the TG orientation, although some events also aligned with the YE orientation. The eastern cluster, located between Tawara Sone and Kappa Sone, was provisionally concentrated near a depth of ~ 20 km, but the latest reanalysis has relocated most events to a depth of ~ 10 km and closer to Kappa Sone (ERC, 9 July 2025). The eastern cluster, the main body of the seismic swarm, is interpreted to reflect activity within the lower crust. Although potentially linked at depth, the spatial segregation of the two groups by the graben zone precludes clear identification of direct continuity.

**Fig. 4:**
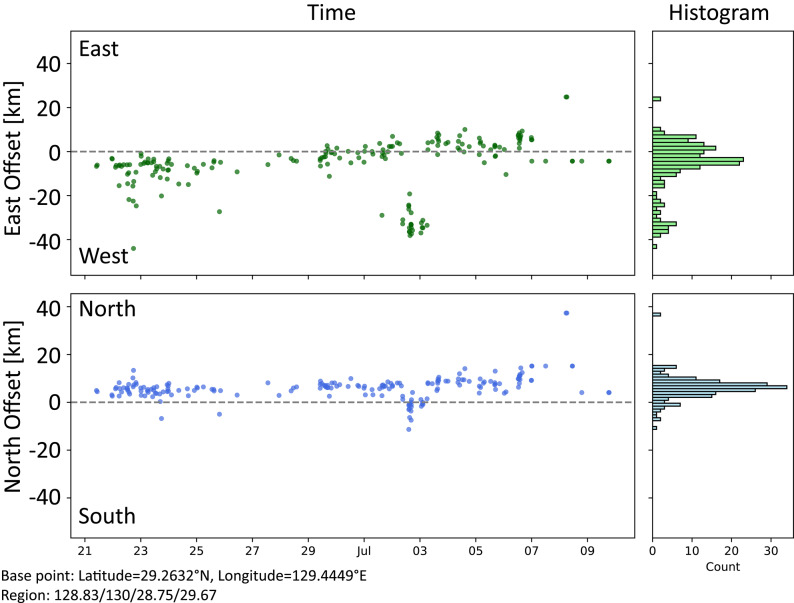
Latitude–time plots and longitude–time plots illustrating the spatiotemporal evolution of the 2025 seismic swarm, with corresponding histograms showing spatial distributions. Left panels show time series of east–west (top) and north–south (bottom) offsets of hypocenters relative to a fixed base point (29.2632°N, 129.4449°E). Right panels show the spatial distribution of events as histograms projected onto the respective axes.

Following the approach of Hrubcová et al.^36^, we visualized the hypocenter migration during the 2025 seismic swarm using the F-net catalog (Fig. 4). This analysis highlighted that the 2025 swarm was not only the most intense in terms of event counts, but it also exhibited a distinctive spatiotemporal evolution. The eastern swarm progressively migrated northeastward (Fig. 4). This pattern reflected a sequential activation of fault segments along the YE-oriented extensional system (Fig. 3).

Because of the limitations of the current seismic observation network, the spatial resolution of hypocenter locations is preliminary.

#### Past swarm sequences and the 2025 event

**Fig. 5:**
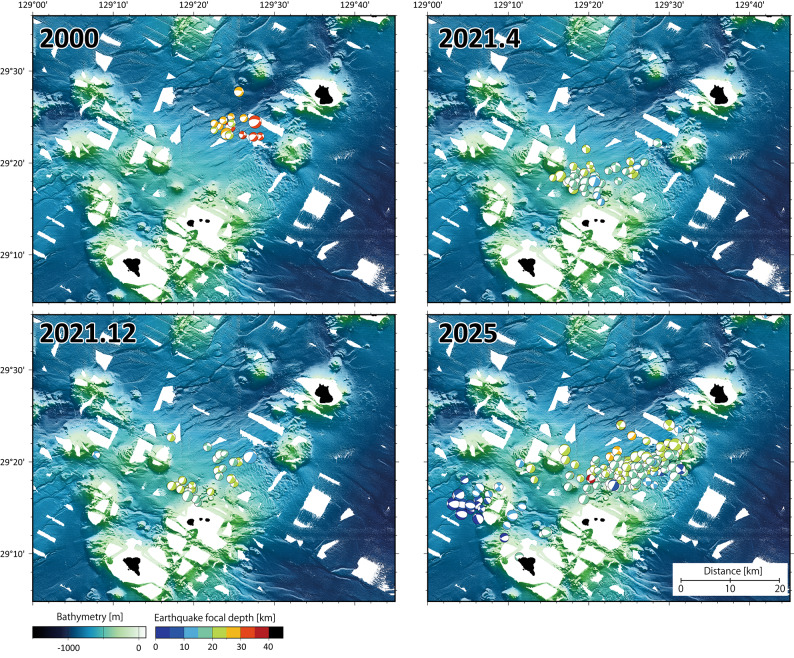
Comparative spatial distribution and focal mechanisms of seismic swarms in the Tokara Gap region (upper left, 2000; upper right, April 2021; lower left, December 2021; and lower right, 2025). Each panel shows epicenters and focal mechanism solutions (F-net catalog^3^ plotted over compiled multibeam bathymetry^9^.

Within the recent swarm event in the Tokara Gap, we compared the spatial distribution and focal mechanisms of four swarm episodes: those in 2000, April 2021, December 2021, and 2025 (Fig. 5). The 1995 swarm is noted for context only. Because the sparse seismic network at that time made its hypocenter locations highly uncertain, it was not included in the comparisons. The 2000 swarm occurred slightly farther north, and its foci were deeper, but with the caveat that the accuracy of the hypocenter locations was limited by the sparse seismic network at the time.

Both the April and December 2021 swarms were tightly concentrated along the eastern part of the arc, and their foci corresponded to the YE-oriented extensional lineament. Focal mechanism solutions from these events consistently indicated an ENE–WSW extension of the fault, in agreement with the prevailing regional tectonic regime.

The 2025 swarm was distinct because it comprised two spatially separated clusters and included a significantly larger number of events than earlier sequences. This dual-cluster pattern, characterized by activity to both the east and west, marked a departure from the simpler distribution observed in past swarms and indicated a more complex fault activation involving both the YE and TG systems.

#### Detailed subsurface structure around swarms

**Fig. 6:**
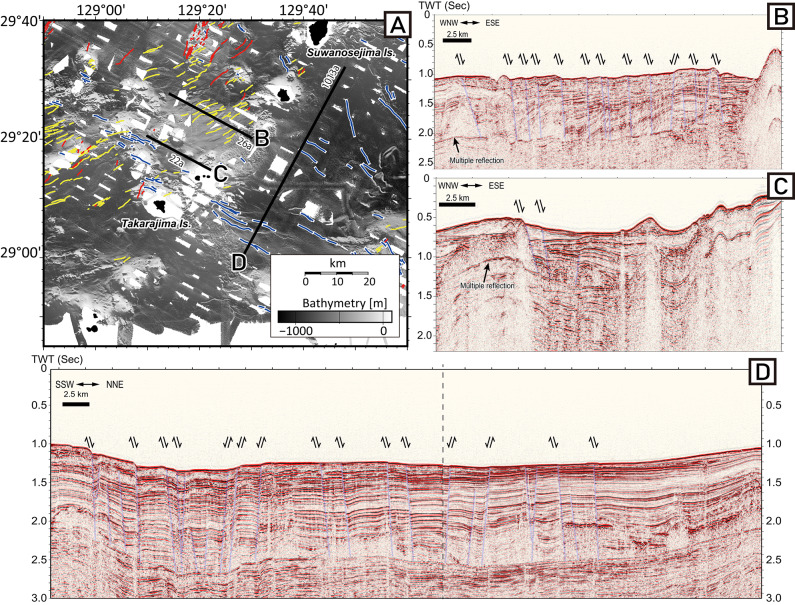
Subsurface structure around the seismic swarm area. (**A**) Index map showing the locations of multichannel seismic profiles across the swarm zone between the islands of Akusekijima and Takarajima. Seismic profiles along (**B**) Line 26a [29.3357°N, 129.5033°E to 29.4620°N, 129.2348°E], (**C**) Line 22a [29.2465°N, 129.3705°E to 29.3438°N, 129.1626°E], and (**D**) Line 1013a [29.5366°N, 129.8007°E to 29.0083°N, 129.4753°E]. Vertical axis is two-way travel time (TWT). A representative upper-crustal velocity of 1500–1800 m/s implies that 1 s on the vertical axis of the seismic profiles corresponds to an estimated depth of ~ 0.75–0.9 km. Due to the survey design, clearly identifiable subsurface structures were limited to depths of ~ 1–2 km Arrows indicate active normal faults reaching the shallow subsurface.

Survey lines were laid out across a Quaternary volcanic front between Kuchino-Erabujima Island and Akusekijima Island. Cross-arc lines (WNW–ESE direction) were spaced at ~ 3.7-km intervals, and parallel-arc lines (NNE–SSW) were spaced at ~ 7.4-km intervals (Fig. 6A; note that, due to the survey design, clearly identifiable subsurface structures were limited to depths of ~ 1–2 km).

High-resolution MCS profiles (Fig. 6B–D) revealed a dense array of normal faults within thick sedimentary sequences that formed a characteristic two-directional rift system between Takara-jima Island and Akuseki-jima Island. This area coincided with intense earthquake swarm activity and was broadly consistent with the observed fault plane solutions.

Along the YE lineament (Fig. 6B), normal faults dipped predominantly eastward, but some faults dipped westward. The more complex fault pattern of the TG lineament (Fig. 6D) included both northward- and southward-dipping faults. In both areas, normal faults could be traced continuously to depths of ~ 1 km below the seafloor and clearly displaced thick surface sedimentary layers. The display of concave geometries by intra-reflection horizons indicated ongoing subsidence and active faulting along both lineaments.

In the swarm center (Fig. 6C), a mound-shaped, stratified sedimentary body characterized by weakly internal reflectors overlaid high-amplitude reflections that were likely of volcanic origin. These bodies were offset by faults linked to the YE system and supported ongoing tectonic activity.

#### Water column imaging and plume activity

Minami et al.^11^ and Wen et al.^35^ have reported hydrothermal plumes in this region (Fig. 3). We analyzed the water column data from cruise KH-22-2 to assess the potential hydrothermal response to the 2021 seismic swarm. Sampling was conducted between 29 January and 5 February 2022, approximately six weeks after the M6.1 earthquake on 17 December 2021. Results confirmed the presence of additional plumes in the broader region (Fig. 3A).

No plume signals were detected above the main seismic swarm area, which includes the small knolls north of Kodakara-jima Island (around 29°17′N, 129°22′E). The lack of plume signals suggested either minimal hydrothermal discharge or highly transient activity detectable only just after seismic events.

### Deeper crustal constraints from potential field data

**Fig. 7:**
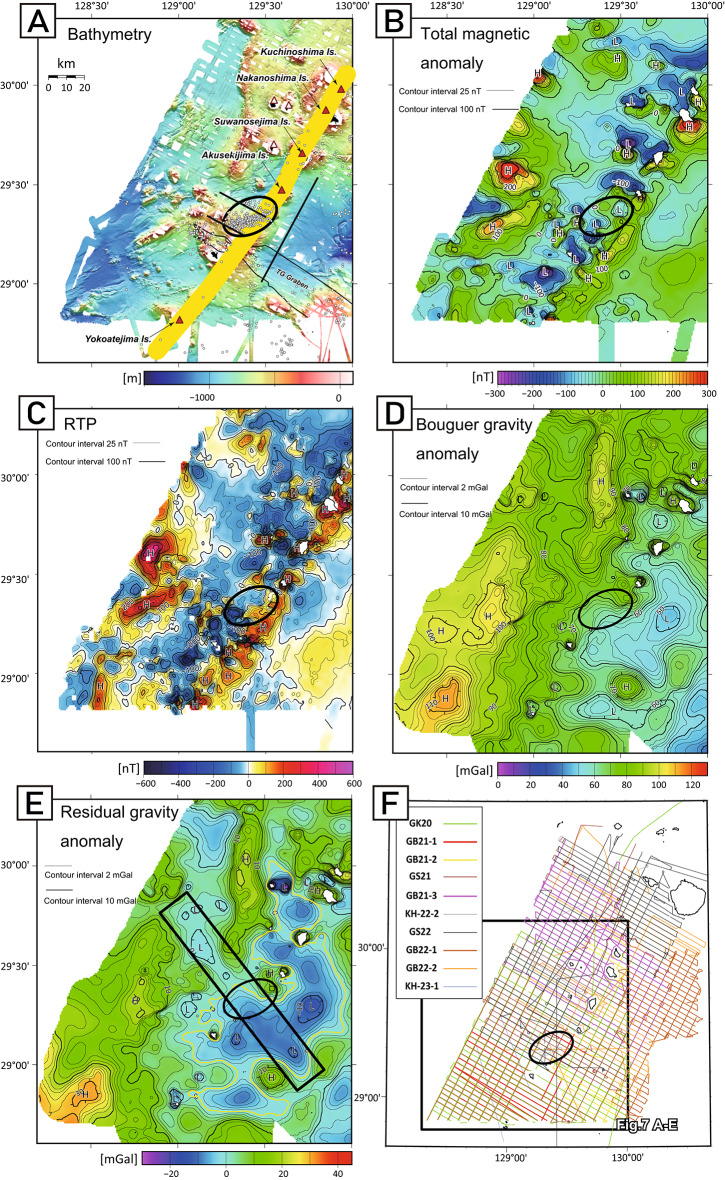
Geophysical maps around the Tokara Islands. (**A**) Bathymetric map with a grid interval of 20 m. The volcanic front is highlighted in yellow. The locations of multichannel seismic profiles shown in Fig. 6 are indicated by bold black lines, and graben structures reported by Minami et al.,^11^ are shown with thin black lines. The primary swarm area in 2025 is delineated by a black oval. White circles indicate the hypocenters of earthquakes from the 2025 seismic swarm. (**B**) Magnetic anomaly map with a grid interval of 0.5 km. Contour intervals are 25 nT (gray lines) and 100 nT (black lines). (**C**) Reduced-to-pole magnetic anomaly map with a grid interval of 0.5 km. Contour intervals are 25 nT (gray lines) and 100 nT (black lines) (**D**) Bouguer gravity anomaly map with a grid interval of 1 km. Contour intervals are 2 mGal (gray lines) and 10 mGal (black lines). (**E**) Residual Bouguer gravity anomaly map obtained by removing long-wavelength components deeper than 20 km from (**D**). The grid interval is 1 km, with contour intervals of 2 mGal. The black rectangle outlines a NW–SE-trending belt of negative residual gravity anomalies within the swarm zone. The yellow contour line denotes a broader negative Bouguer gravity anomaly distributed around the islands. (**F**) Track lines of ship around the Tokara Islands, with each cruise shown in a different color.

Figure 7B,C illustrate magnetic anomaly results. Figure 7A provides an overview of the regional bathymetry, and Fig. 7F summarizes the ship track lines used in this study. To simplify the interpretation of the magnetic data, anomalies were reduced to the pole (RTP) (Fig. 7C) to position them directly above their geological sources^37^. The RTP was implemented with ‘grdredpol’ in Generic Mapping Tools version 6 (GMT6).

Previous studies based on conventional total-field magnetic anomalies have shown that subaerial volcanoes along the Quaternary volcanic front, including the islands of Suwanosejima, Nakanoshima, and Akusekijima, exhibit prominent, short-wavelength, dipolar magnetic anomalies with narrow contour spacing consistent with their recent eruptive activity^8^. These volcanoes typically display dipolar patterns characterized by positive and negative values to the south and north, respectively. We observed similar patterns (Fig. 7B, C). That the magnetic anomalies were generally subdued east of the volcanic front suggested that there were few shallow crustal magnetic sources, whereas there were markedly more complex magnetic structures on the western side.

Incorporation of newly acquired data into the RTP anomaly map (Fig. 7C) revealed a belt of positive magnetic anomalies along the volcanic front. As in previous work, the distribution of magnetic anomalies west of the front remained highly complex. West of the swarm area, two small dipolar anomalies with wavelengths of ~ 20 km were apparent (Fig. 7B). These anomalies appeared as a single continuous high in the RTP map. This region is also characterized by well-developed knolls and pronounced bathymetric relief consistent with the distribution of magnetic anomalies. Within the swarm area, two distinct, dipolar magnetic anomalies were apparent, with peak values of approximately − 75 to 100 nT and − 125 to 25 nT, respectively. Both anomalies extended over ~ 5 km, and in the RTP map they appeared as part of a continuous positive anomaly aligned with the volcanic front. These short-wavelength magnetic anomalies consisted of small volcanic knolls that were apparent in the bathymetric data (Fig. 5) and suggested that there were localized, shallow, crustal magmatic sources that may have been structurally linked to the swarm activity. The Bouguer gravity anomaly map revealed a relatively low anomaly on the eastern side of the island arc, between the islands of Akusekijima and Tairajima, that corresponded to a sedimentary basin (Fig. 7D). Positive gravity anomalies associated with the OT were widely distributed to the west of the island arc. Positive gravity anomalies were also clearly observed along ridge structures in the northern part of the study area, around 129°25′E, 30°N. In contrast to the magnetic anomalies, there was no apparent correspondence between the Bouguer gravity anomalies and the graben described by Minami et al.^11^.

Because earthquake hypocenters tend to be concentrated near the Moho or at shallower depths, we calculated residual gravity anomalies. We focused on shallow structures by extracting short-wavelength components corresponding to depths shallower than 20 km (Fig. 7E). Results indicated that negative gravity anomalies associated with the basin were more broadly distributed than those revealed by the Bouguer anomalies (yellow contours in Fig. 7E). A NW–SE-trending belt of negative anomalies was clearly apparent (black box in Fig. 7E). This belt-like anomaly did not directly correspond to seafloor topography and was near but slightly offset from the graben described by Minami et al.^11^. The alignment of these swarm earthquake hypocenters along both this graben and the belt of negative gravity anomalies suggested a possible spatial link between the fault structures and the swarm activity.

## Discussion

### Structure and seismic activity

Our multichannel seismic profiles and fault-striation analysis with solutions of the focal mechanism combined with the integrated analysis of high-resolution bathymetry from ten cruises previously presented by Koge et al.^9^ demonstrated that seismicity in the Tokara region is localized within a structurally complex zone where three extensional lineament groups converge. The resemblance of the configuration of these three arms to the geometry of a Ridge–Ridge–Ridge–type triple junction indicates the interaction of multiple extensional systems and production of a characteristic deformation pattern across the arc.

The present study showed that the 2025 seismic swarm was concentrated specifically within the intersection of two dominant lineaments. The WNW–ESE trending TG lineament (orientation N71.74°W; blue shading in Fig. 2B) represents the principal cross-arc extensional structure that cuts across the volcanic front. The ENE–WSW trending YE lineament (orientation N71.30°E; yellow shading in Fig. 2B) is oriented mainly along the arc and forms an oblique, arc-parallel extensional system. The intersection zone, which lies immediately adjacent to the volcanic front, adds a layer of structural complexity and likely plays a key role in focusing swarm activity.

The distribution of hypocenters, the shallow normal faults that extend up to the seafloor, and the presence of small volcanic knolls collectively indicate active extensional deformation within this zone. A NW-SE trending belt of negative residual Bouguer gravity anomalies spatially coincides with part of the distribution of the swarm hypocenter. The implication is that deformation may extend through a substantial portion of the crust. Close agreement between the orientations of the extensional axes derived from the focal-mechanism solutions and the mapped fault trends further highlights the dominant role of these cross-cutting extensional systems in controlling seismic swarm activity.

Previous swarm episodes in the region have been restricted almost entirely to the eastern YE fault zone, but the 2025 event exhibited both greater spatial complexity and a distinct temporal evolution. The 2025 sequence included an additional western cluster within the TG graben (Fig. 5), and the hypocenters in the eastern cluster migrated progressively northeastward over time (Fig. 4). This spatially bifurcated configuration and pattern of temporal migration suggested that fault interactions at the intersection of the TG and YE systems became more dynamically active during the 2025 event. These interactions may have been influenced by increased input of fluids or magma, or by local changes of stress distribution. Given the relatively simple migration trajectory and short timescale of the eastern cluster, low-viscosity fluids or gases, rather than magma, may have primarily triggered the swarm activity. Whether the eastern and western clusters represent a single connected process or two distinct episodes is unclear.

### Swarm seismicity in initial rifting

There have been two estimates of the depth of the 2025 Tokara seismic swarm. The F-net catalog placed the majority of hypocenters near 20 km (Fig. 3), whereas a recent relocation by the ERC (9 July 2025) suggested a concentration around 10 km near Kappa Sone. Both estimates pointed to rupture within the upper to middle crust rather than activity at the continental Moho, which lies at a depth of ~ 25 km^2^.

The consistent crustal concentration of swarm events in the Tokara Gap suggested the presence of a characteristic shallow-to-intermediate seismogenic layer. The close resemblance of this distribution to those observed in other incipient rift systems underscored the similarities between the Tokara swarms and seismic clusters associated with early-stage back-arc rifting.

### Potential trigger: coupling between fluids, magma, and earthquakes

**Fig. 8:**
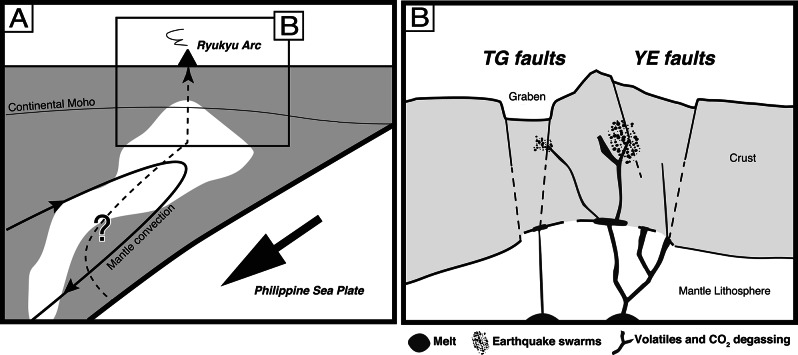
Conceptual models of fluid pathways and seismic swarm generation. (**A**) Schematic diagram illustrating slab dehydration, fluid transport, and crustal melt assimilation processes in the Ryukyu arc setting, originally proposed for the NE Japan arc^38^ and here modified and applied to the Ryukyu arc setting. (**B**) Conceptual image of magma and fluid migration pathways in the lithosphere beneath the Tokara Gap, showing zones of volatile release, deep mantle alteration, and earthquake swarm occurrence. Modified after Lindenfeld et al.^39^ and originally proposed for the Rwenzori region.

Conceptual models of fluid transport originally developed for the northeastern Japan arc have demonstrated how slab-derived fluids ascend through the mantle wedge and contribute to arc magmatism (e.g.^38^) (Fig. 8). This model provides a useful framework for considering volatile transfers beneath volcanic fronts. Two examples of seismic swarm activity provide useful analogies. At the Rwenzori segment, located at the western termination of the East African Rift^39^, seismic swarms occur at depths of 5–16 km, recur episodically, and exhibit normal faulting mechanisms driven primarily by migration of CO_2_-rich fluids. Notably, these swarms occur without surface volcanism and highlight fluid-driven fault activation concentrated at structurally complex intersections. In contrast, repeated seismic swarms off the eastern Izu Peninsula of Japan have been interpreted as responses to shallow dyke intrusions within an extensional tectonic regime. This interpretation has been supported by tiltmeter, global positioning system, and seismic data (e.g.,^40–43^).

The 2025 Tokara swarm exhibited a mixed character. Bathymetric data showed only minor volcanic edifices, yet localized, short-wavelength magnetic anomalies (Fig. 7B) suggested possible shallow crustal magmatic activity. In the RTP map (Fig. 7C), these anomalies appeared as part of a continuous positive magnetic belt along the volcanic front. The implication was that the shallow magmatic activity beneath the swarm area may be structurally connected to, or even sourced from, the volcanic front. Geochemical signatures from the island of Kodakarajima^35^ indicated strong inputs of mantle volatiles and underscored the significant role of mantle-derived fluids beneath the volcanic front. Preliminary data from the Geospatial Information Authority of Japan (GSI, press releases on 4 July 2025 and 18 July 2025)^44,45^ indicated rapid and contrasting crustal deformation around the island of Takarajima during the ongoing seismic swarm. Between 1 June and 4 July 2025, Takarajima moved ~ 3.7 cm toward the south-southeast. Shortly thereafter, the displacement reversed direction, and ~ 0.8 cm of west-northwestward movement was recorded during 14–16 July 2025. This abrupt change could have reflected the influence of pressurized fluids, although the possibility that two separate swarm episodes occurred in close succession cannot be excluded.

A water-column survey conducted about six weeks after the December 2021 swarm detected no hydrothermal anomalies directly above the swarm hypocenters. The absence of hydrothermal anomalies may indicate either that localized or short-lived activity was not captured during the survey or that fluids were not involved at shallow levels.

These observations confound determination of whether the intrusion involved deep crustal fluids, magmatically derived fluids, magma itself, or a combination of these components. Integration of geological, geophysical, and geochemical evidence emphasizes that the 2025 Tokara swarm was influenced by a structural framework capable of channeling volatile-rich fluids and potentially magma into the upper crust. This configuration likely facilitated the ascent of such materials and may have controlled spatial clustering of swarm activity. Such a configuration would provide interconnected pathways that could facilitate transport of fluid and magma beneath the Tokara Gap (Fig. 8). This possibility may also explain the spatial clustering of swarm activity. This configuration will be a key target for future monitoring and hazard assessment.

## Conclusion

We used an integrated synthesis of tectonic setting, structural geology, and multidisciplinary datasets to refine understanding of Tokara tectonics and examine the mechanisms that have generated seismic swarms, including the unprecedented 2025 event. The results showed that the swarm activity in this nascent back-arc rift could not be captured by a single-process model. Instead, the swarm arose from the coupled action of deep crustal fluids, localized magmatic inputs, and structure-controlled stress near the volcanic front.

The Tokara Gap region may represent an incipient RRR triple junction. In such a setting, whether crustal extension is accommodated primarily by plume-related magmatism or by tectonically driven fluid pathways has implications for the evolution of triple junctions worldwide. While the relative contributions of these processes at Tokara are unclear, our results highlight the critical role of complex fault intersections in providing pathways for ascent of both fluids and magma.

Continued geophysical, geochemical, and geological investigations will be necessary to refine this model and improve seismic hazard assessments in tectonically and volcanically active regions.

## Methods

This study made use of key methodologies used in marine geological surveys, including multibeam echosounder (MBES) bathymetry, water column imaging, multichannel seismic (MCS) reflection surveys, and magnetic anomaly measurements (Table S1). Because the methods of data acquisition and detailed data-processing procedures in each survey have been described in previous reports^34,8,46^, only brief summaries are provided below. In addition, we describe the procedure used to calculate Bouguer gravity anomalies based on the acquired bathymetric data and the derivation of residual gravity anomalies.

### Multibeam echosounder (MBES) surveys

We compiled bathymetric data from nine seafloor geological mapping cruises (see Table A1 in the Appendix of^9^). Among these cruises, the KH-22-2 (R/V *Hakuho-Maru*) cruise, which was conducted in 2021, specifically focused on the earthquake swarm area and is therefore highlighted here (details in Tamura^47^). The Kongsberg EM124 system onboard the *Hakuho-Maru* was integrated with a Seapath 380-5 + system for positioning, heave correction, and heading estimation. The observation frequency ranged from 10.5 to 13.5 kHz, with a maximum of 512 beams. Conductivity-temperature-depth (CTD) profiles were collected using expendable CTD sensors and eXpendable Bathy Thermograph (XBT) probes and were merged with deep-water velocity profiles from the NOAA Levitus dataset^48^ to correct for the structure of sound velocity. The EM124 also enabled simultaneous acquisition of backscatter intensity and water column data. Given the potential involvement of hydrothermal fluids and volcanic processes, water column data were recorded during this cruise. Preliminary visual inspection revealed acoustic anomalies in the water column recordings.

### Multi-channel seismic (MCS) reflection surveys

MCS reflection surveys were conducted during seven cruises as part of the marine geological mapping program (GB21-1, GB21-2, GB21-3, GS22, GB22-1, and GB22-2).

The specifications and cruise-specific details have been reported in Ishino et al.^34,49,50^. Most of the geological mapping cruises used a portable MCS system described below. Technical details for each cruise are available in previous reports, including Ishino et al.^34^.

Survey lines were laid out across a Quaternary volcanic arc between Kuchinoerabu-jima Island and Akuseki-jima Island. Orthogonal lines (WNW–ESE direction) were spaced at intervals of ~ 3.7 km and arc-parallel lines (NNE–SSW) at intervals of ~ 7.4 km.

The seismic source was a single GI gun (Sercel) (Generator: 250 cu. in.; Injector: 105 cu. in.). The receiving system was a GeoEel Solid digital streamer (Geometrics) with 16 channels spaced at 12.5-m intervals. The source was fired every 6 s while maintaining a vessel speed of ~ 8 knots. The distance between shots was therefore ~ 25 m, and the common midpoint (CMP) spacing was ~ 6.25 m. Data were recorded with a CNT-2 (Geometrics) system and saved in SEG-D format. After conversion to SEG-Y format, basic processing was applied to the data using Seismic Processing Workshop software (version 4.0; Parallel Geoscience Corporation; https://parallelgeo.com/). Data processing included geometry editing, band-pass filtering, spherical divergence corrections, deconvolution to suppress short-period multiples, and velocity analysis. Normal moveout (NMO) corrections were applied, followed by CMP stacking with four traces to enhance the signal-to-noise ratio. With a dominant frequency of 35 Hz and a P-wave velocity of 1500 m/s, the vertical resolution of the final seismic sections was estimated to be ~ 10 m based on Rayleigh’s quarter-wavelength rule (e.g. SEGJ^51^). Note that our interpretations rely on 2D seismic profiles.

### Magnetic anomalies

For the GB21-2, GB21-3, GB22-1, GB22-2, and GS22 cruises, total field magnetic data were acquired using a towed cesium magnetometer (Model G-882, Geometrics) deployed ~ 300 m astern of the vessel to reduce ship-induced magnetic noise. Data processing included cable length correction, diurnal variation correction using Kakioka Observatory data^52,53^, and subtraction of the 14th edition of the International Geomagnetic Reference Field (IGRF-14; IAGA^54^) to derive magnetic anomalies.

For the GK20 and GB21-1 cruises, vector magnetic data were obtained using a three-component fluxgate magnetometer (Model SFG-2009, Terra Technica) from which total field values were calculated after applying correction coefficients. As we did with the cesium magnetometer data, we subtracted the International Geomagnetic Reference Field (IGRF-14; IAGA^54^) before gridding. However, because of its lower sensitivity compared to the cesium magnetometer, we did not apply a diurnal correction. Instead, we applied detrending and leveling corrections using regional magnetic anomaly data from the Magnetic Anomaly Map of East and Southeast Asia, Revised Version 3rd Edition^55^. Details of the vector magnetic data processing procedures have been summarized by Koge et al.^8^.

After these corrections had been applied, all magnetic datasets were merged and interpolated onto a uniform 1-min spatial grid. To simplify the interpretation of the magnetic data, anomalies were reduced to the pole (RTP) so that they were positioned directly above their causative sources^37^. In this study, the RTP transformation was performed with ‘grdredpol’ in GMT6^12^ and IGRF-14 (declination − 6.809°, inclination 43.477°, elevation 0 m).

### Gravity anomaly

Because only the R/V *Hakuho-Maru* was equipped with a marine gravimeter and because its spatial coverage was limited, we used the nationwide dataset compiled in the Gravity Database of Japan (GSJ, AIST^56^) for the absolute gravity and free-air gravity anomalies. This database contains 1,708,881 gravity observation points around Japan (470,814 on land and 1,238,067 offshore) that include measurements collected by multiple research institutions, governmental geological surveys, and private-resource exploration projects. From this comprehensive dataset, we generated a uniform 500-m, free-air anomaly grid for subsequent processing.

Both Bouguer and terrain corrections were computed using the FA2BOUG program^57^ assuming crustal and water densities of 2670 and 1030 kg m⁻³, respectively. The corrections were performed on a 0.5-arcmin grid. The FA2BOUG program calculates Bouguer and terrain corrections by integrating topography at multiple spatial resolutions after dividing the calculation area into several computational zones with different grid spacings. This multiscale approach enables the combination of fine local topography with coarser global models and is particularly effective for mixed land–sea regions, including islands because it does not assume any subsurface continuation below the seafloor. For the topographic input, we used both global and local datasets: global topography from ETOPO1 (^58^; 1-arcmin resolution) and high-resolution bathymetry (20-m grid) from multibeam surveys^9^. Gravity corrections were conducted only in marine regions because terrestrial areas were outside the scope of this study. The resulting Bouguer anomaly grid was output at 30-arcsecond resolution and subsequently smoothed and resampled to a 100-m grid using Generic Mapping Tools (GMT 6^12^) to reduce block-shaped artifacts and enhance field continuity. The result was a smoothed Bouguer anomaly map with an effective resolution of ~ 100 m, optimized for identifying short-wavelength gravity variations associated with shallow crustal and volcanic structures in the Tokara region.

The Mantle Bouguer Anomaly (MBA) can provide valuable insights into density contrasts at the crust–mantle boundary and can help distinguish shallow crustal or sedimentary effects from deeper mantle processes. However, the MBA was not computed in this study because the Tokara region lacks structural simplicity and reliable subsurface constraints required for such long-wavelength corrections. Crustal thickness varies markedly across the arc–back-arc transition^21^, and because middle Miocene forearc alkaline magmatism (e.g.,^59^) suggests that heterogeneous crustal fragments have been incorporated into the forearc lithosphere, it is difficult to define a laterally consistent density structure. In addition, the sedimentary cover is both thick and laterally heterogeneous. The presence of several kilometers of continent-derived deposits in the back-arc basins (e.g.,^34,49,50^) contrasts sharply with the relatively uniform crustal settings where MBA corrections are typically applied. Because of these confounding factors, applying an MBA correction would likely introduce artifacts comparable to, or even larger than, the genuine long-wavelength signal.

Instead, to emphasize shallow crustal structures while minimizing deeper slab-related components, we computed residual gravity anomalies with ‘grdfft’ (GMT 6^12^). The procedure involved applying a 2-D Fast Fourier Transform (FFT) to the Bouguer anomaly grid, filtering the spectrum in the frequency domain, and reconstructing the spatial field by inverse FFT. We first extracted the long-wavelength component using a low-pass filter corresponding to structures deeper than ~ 20 km, and then we subtracted that field from the Bouguer anomaly to obtain the short-wavelength residual gravity. Although the regional Moho lies at a depth of ~ 25 km based on P-wave models^29^, we adopted a slightly shallower cutoff to suppress potential slab influence and to ensure that the extracted anomalies predominantly reflected shallow crustal variations.

### Focal mechanism

All focal mechanisms in this paper were obtained without modification from the NIED F-net moment tensor catalogue^60^, which provides broadband waveform-inversion solutions for regional earthquakes using a one-dimensional velocity structure model. Only solutions that satisfy the internal quality criterion based on variance reduction are released as stable mechanisms.

## Supplementary Information

Below is the link to the electronic supplementary material.

Supplementary Material 1

## Data Availability

Marine geophysical data from the R/V **Bosei-Maru** , T/S **Shinyou-Maru** , and R/V **Kaiyo-Maru No. 2** were collected as a part of the GSJ (AIST) geological mapping program. In accordance with the policies of this program, raw geophysical datasets are made publicly available after publication of the corresponding geological map products. Cruise reports and survey documentation are publicly accessible. Bathymetric products derived from these surveys are already available as GeoTIFF files in the Appendix of Koge et al. (2025). Data from the R/V **Hakuho-Maru** are managed by the Japan Agency for Marine-Earth Science and Technology (JAMSTEC), and a portion of the datasets and cruise reports are publicly available through the JAMSTEC data portal.
