# Supplementary material for: Tectonics of earthquake swarms in the Tokara Gap of northern Ryukyu Arc based on marine geological and geophysical surveys
Source: Sci Rep. 2026 Apr 18;16:22455. doi: 10.1038/s41598-026-41371-z (PMC13376354; doi:10.1038/s41598-026-41371-z)
Supplement: Supplementary file 1 — Supplementary Material 1 [file 41598_2026_41371_MOESM1_ESM.pdf]

Cruise Name	Ship	Date	Survey items			
			MBES	MCS	Magnetometer	Gravimeter
GK20	R/V Kaiyo-maru No. 2	2020 Nov. – Dec. (19 days)	EM 302, Kongsberg	-	Three-component fluxgate magnetometer	-
GB21-1	RTV Bosei-maru	2021 Feb. – Mar. (29 days)	EM 302, Kongsberg	One G.I. gun and 200 m (16 ch) streamer system	Three-component fluxgate magnetometer	-
GB21-2	RTV Bosei-maru	2021 Jul. (5 days)	EM 302, Kongsberg	One G.I. gun and 200 m (16 ch) streamer system	Towed cesium magnetometer	-
GS21	T/S Shinyo-maru	2021 Oct. (20 days)	Seabeam3050, ELAC SONAR GmbH	One G.I. gun and 200 m (16 ch) streamer system	Towed cesium magnetometer	-
GB21-3	RTV Bosei-maru	2021 Oct. – Nov. (34 days)	EM 302, Kongsberg	One G.I. gun and 200 m (16 ch) streamer system	Towed cesium magnetometer	-
KH-22-2	R/V Hakuho-maru	2022 Jan. – Feb. (8 days)	EM 124, Kongsberg	-	※Towed proton magnetometer	※Shipboard gravimeter (Model D-004, LaCoste & Romberg, ZLS)<br>※Portable gravimeter (CG-5 AUTOGRAV, Scintrex)
GS22	T/S Shinyo-maru	2022 May – Jun. (19 days)	Seabeam3050, ELAC SONAR GmbH	One G.I. gun and 200 m (16 ch) streamer system	Towed cesium magnetometer	-
GB22-1	RTV Bosei-maru	2022 Jul. (19 days)	EM 302, Kongsberg	One G.I. gun and 200 m (16 ch) streamer system	Towed cesium magnetometer	-
GB22-2	RTV Bosei-maru	2022 Nov. (26 days)	EM 302, Kongsberg	One G.I. gun and 200 m (16 ch) streamer system	Towed cesium magnetometer	-
KH-23-1	R/V Hakuho-maru	2023 Jan. – Feb. (10 days)	EM 124, Kongsberg	※AORI system	※Towed proton magnetometer	※Shipboard gravimeter (Model D-004, LaCoste & Romberg, ZLS)<br>※Portable gravimeter (CG-5 AUTOGRAV, Scintrex)

※ indicates data were acquired but not included in this work.

Table S1 | Summary of cruises
